# Supplementary figures and images for: Characterization of a human monoclonal antibody generated from a B-cell specific for a prefusion-stabilized spike protein of Middle East respiratory syndrome coronavirus
Source: PLoS One. 2020 May 8;15(5):e0232757. doi: 10.1371/journal.pone.0232757 (PMC7209324; doi:10.1371/journal.pone.0232757)

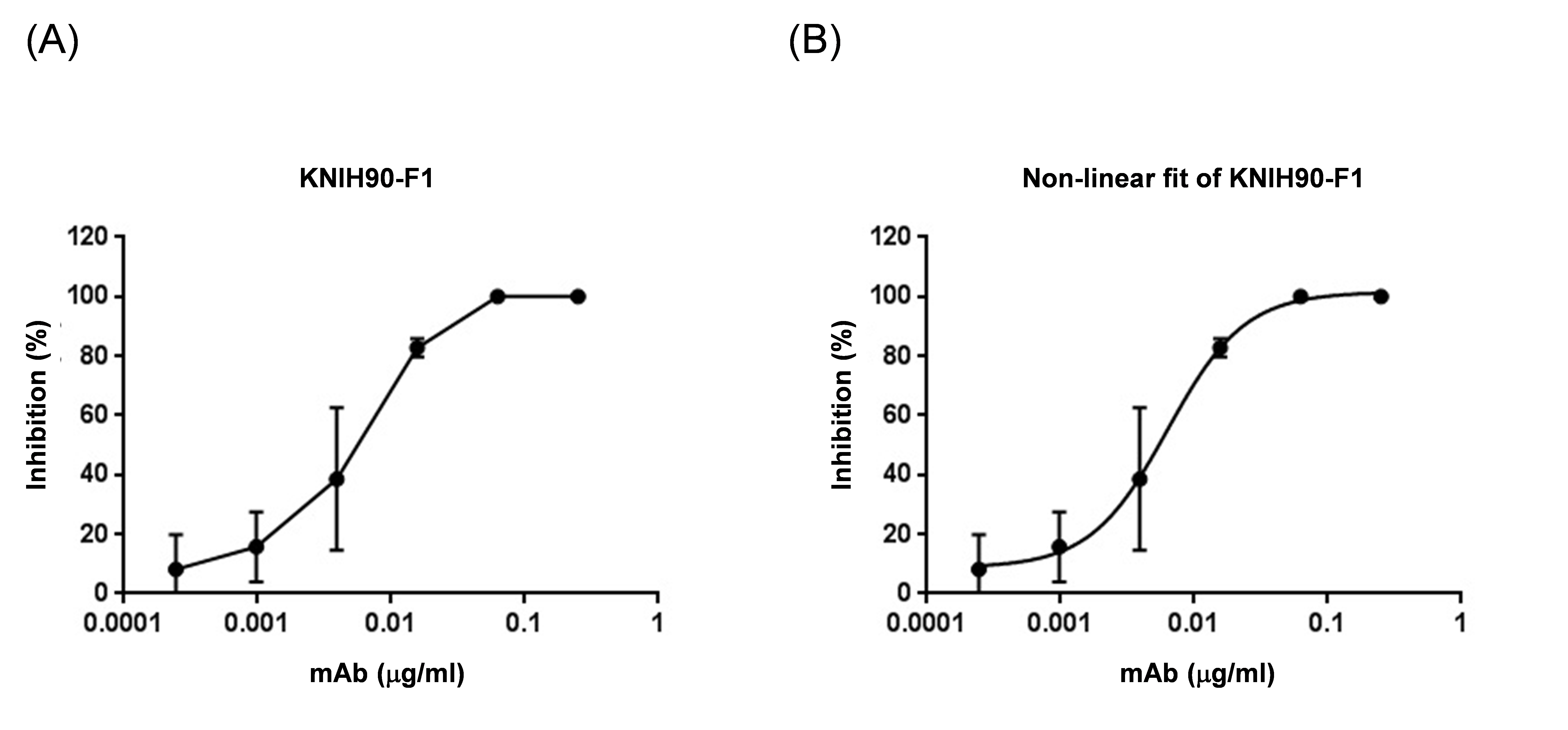

Supplement: S1 Fig — Neutralization potency of the RBD-specific mAb KNIH90-F1 was evaluated by a PRNT assay using low mAb concentrations (0.00025–0.25 μg/ml). Percent inhibition by the mAb was determined by comparison with untreated cells (A). The IC50 value was determined by nonlinear regression curve fitting (B). Each experiment was performed in triplicate, and data represent the mean of each experiment with standard errors. (TIF) [file pone.0232757.s001.tif]

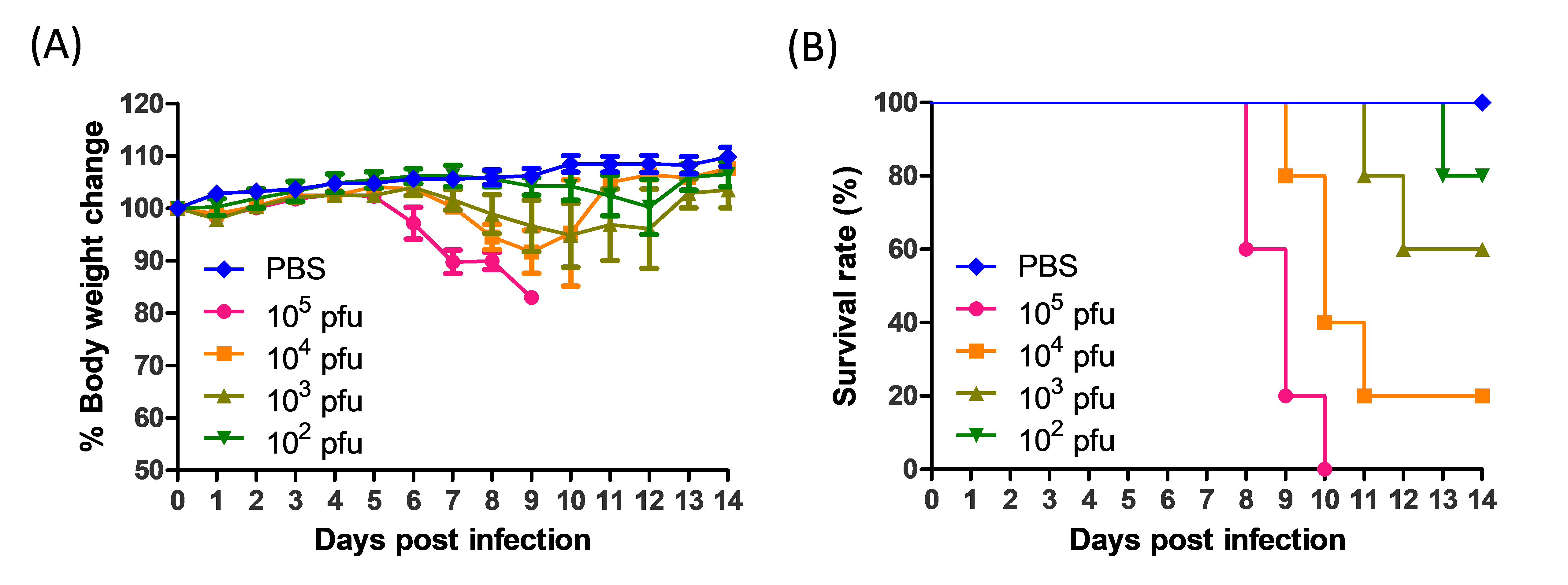

Supplement: S2 Fig — (A) Body weight loss curve of 10-week-old male C57BL/6N-hDPP4 mice (n = 5 per group) infected with different doses of MERS-CoV KNIH-002. The error bar indicate the standard error of the mean. The weight loss and survival were monitored for 14 days. (TIF) [file pone.0232757.s002.tif]

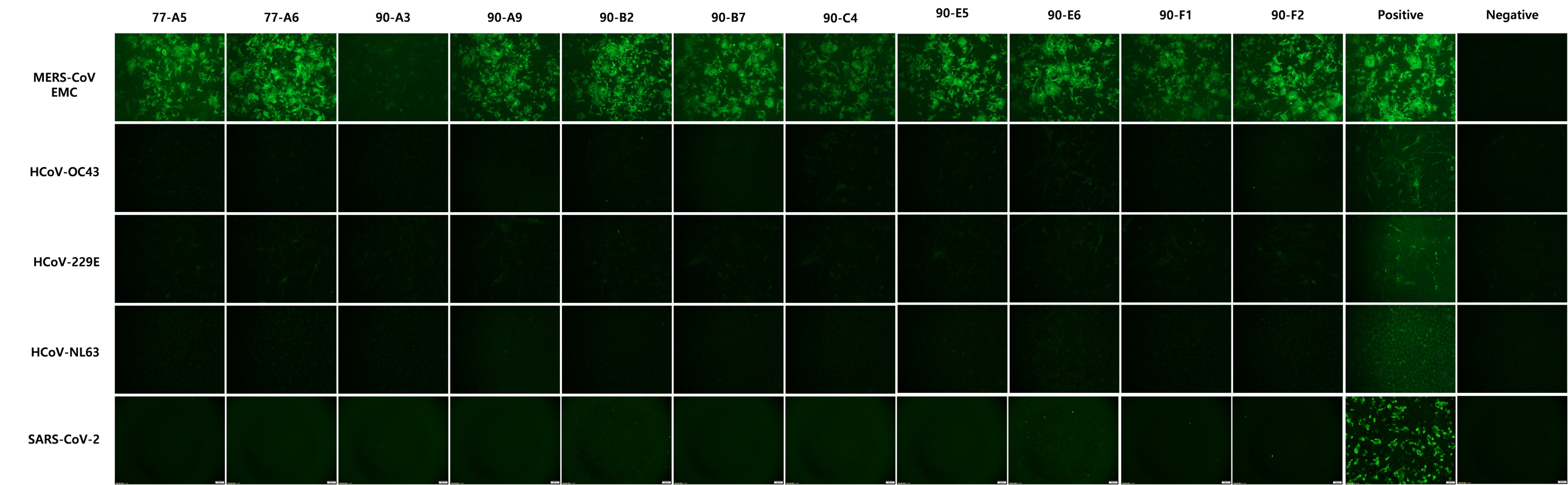

Supplement: S3 Fig — IFA was performed as described previously [27]. Briefly, Vero cells were infected with MERS-CoV Erasmus (EMC) strain and SARS-CoV-2 isolated from a Korean patient [50]. MRC5 cells were infected with human coronaviruses (hCoV) OC-43 strain (ATCC, Manassas, VA, USA) and 229E strain (ATCC). LLC-MK2 cells were infected with hCoV NL63 strain (CN061/14). After the cytopathogenic effect appeared in the infected cells, the cells were fixed with 70% methanol in PBS. Then, human anti-MERS-CoV antibodies were incubated with the Vero cells infected with MERS-CoV. As positive controls, a rabbit polyclonal anti-MERS-CoV spike protein antibody (Sino biological Inc., Beijing, China) for MERS-CoV, a mouse anti-229E coronavirus nucleoprotein OC-43 antibody (MERCK, Darmstadt, Germany) for HCoV-229E, a mouse anti-coronavirus antibody, hCoV OC-43 (LifeSpan BioScience, Seattle, WA, USA) for HCoV-OC43, a rabbit polyclonal anti-hCoV-HKU1 spike protein antibody (Sino Biological Inc., Beijing, China) for hCoV-NL63, a human serum of a Korean SARS-CoV-2 convalescent person were also incubated. The IFA performed without the positive primary antibodies (described above) were used as negative controls. The secondary antibodies, Fluorescein isothiocyanate (FITC) -conjugated goat anti-human-IgG (Abcam, Cambridge, U.K.), FITC-conjugated goat anti-rabbit-IgG (Abcam, Cambridge, U.K.), and FITC-conjugated goat anti-mouse-IgG (Jackson Immunoresearch, West Grove, PA, USA) were added to the cells and incubated. The cells were observed, and images were obtained using a fluorescence microscope. (TIF) [file pone.0232757.s003.tif]
